# Using Functional Education Appliance on One Patient with Class III Malocclusion in Mixed Dentition: A Case Report

**DOI:** 10.3390/children12091219

**Published:** 2025-09-11

**Authors:** Chun-Yuan Chiu, Shang-Wen Chiu, Chung-Hsing Li

**Affiliations:** 1 Department of Dentistry, Tri-Service General Hospital, Neihu, Taipei 114202, Taiwan; 2 School of Dentistry, College of Oral Medicine, National Defense Medical University, Neihu, Taipei 114201, Taiwan; 3 Graduate Institute of Dental Science, College of Oral Medicine, National Defense Medical University, Neihu, Taipei 114201, Taiwan

**Keywords:** prefabricated myofunctional appliance, Class III malocclusion, interceptive orthodontic treatment

## Abstract

**Highlights:**

**What are the main findings?**

- Prefabricated myofunctional appliances are effective options for managing skeletal Class III malocclusion in pediatric patients with vertical mandibular growth patterns.
- Treatment with prefabricated myofunctional appliances has a three-dimensional effect: sagittal, transverse, and vertical.

**What is the implication of the main finding?**

- Prefabricated myofunctional appliances offer an optimal force/moment mechanism in treating Class III malocclusion in pediatric patients.
- Treatment outcomes result from appliance-produced skeletal and dental effects, and craniofacial growth (including a short-term acceleration period).

**Abstract:**

Background/objective: Class III malocclusion is a relatively common clinical problem among Asian patients, which is caused by skeletal discrepancies and involves complex factors. In growing patients, early intervention with myofunctional appliances can help correct jaw relationships more effectively. This case report presents the use of prefabricated myofunctional appliances (EF and MRC) to address Class III malocclusion through growth modification. Case description: A 9-year-old girl was diagnosed with skeletal Class III and a complete anterior crossbite. She underwent treatment with the prefabricated myofunctional appliances, worn during sleep and an additional two hours during the day for 51 months. The outcomes resulted from a combination of skeletal and dental changes, including a decrease in skeletal discrepancy (ANB: −5° to −2°), upper incisor proclination (U1 to SN: 112.5° to 123°), uprighting of the lower incisor (L1 to MP: 93.5° to 90°), and an increase in cranial flexure angle (123° to 125°) with a vertical mandibular growth pattern. The treatment improved facial profile, reduced skeletal discrepancy, corrected the anterior crossbite, and enhanced interdigitation. Conclusions: Prefabricated myofunctional appliances are effective options for managing skeletal Class III malocclusion in pediatric patients with vertical mandibular growth patterns, producing favorable skeletal and dentoalveolar changes.

## 1. Introduction

Class III malocclusion is one of the most challenging conditions in orthodontic practice, characterized by a disharmonious relationship between the maxilla and mandible, often presenting with an anterior crossbite and a concave facial profile. It demonstrates notable ethnic variations in prevalence, occurring with relatively high frequency in Asian populations, where the incidence in Chinese and Japanese populations ranges between 4% and 14%, compared to only 1% to 4% in Caucasians [1]. The etiology of Class III malocclusion is multifactorial, with possible causes including deficient maxillary growth, excessive mandibular horizontal growth, deficient mandibular vertical growth, or combinations thereof [2]. Additionally, a genetic predisposition and environmental factors, such as oral habits and airway obstruction, may contribute to its development [3,4]. In juvenile Class III jaw discrepancies, anterior crossbite is a common clinical feature [5]. If left untreated, it often leads to functional complications, esthetic concerns, and psychosocial impacts that can significantly affect a patient’s quality of life.

Appropriately timed growth modification treatment during the mixed dentition phase can effectively and efficiently treat disproportionate jaw growth and improve occlusion and facial esthetics. Early intervention aims to normalize the skeletal relationship between the jaws, eliminate functional interferences, and potentially reduce or eliminate the need for orthognathic surgery in adulthood. Various treatment modalities, including functional appliances, facemasks, and chin cups, have been proposed to treat skeletal Class III in children with mixed dentition [1]. However, conventional Class III orthopedic treatments often involve the use of extraoral appliances, whose primary drawback is their unesthetic appearance, which may reduce patients’ compliance. The success of early treatment is critically dependent on accurately assessing the patient’s growth potential and selecting the optimal timing for intervention.

The cervical vertebral maturation (CVM) method has been widely used as a valuable diagnostic tool for assessing skeletal maturity by evaluating the morphology of the second to fourth cervical vertebrae on lateral cephalometric radiographs, thereby avoiding additional radiation exposure. Recent studies have reinforced the validity of this method as a reliable and non-invasive skeletal maturity indicator, highlighting gender- and age-related differences in growth patterns. Vaida et al. (2019) analyzed 215 orthodontic patients and reported a significant correlation between dental age and bone age, with patients who had higher dental age values also exhibiting higher bone age values; females showed a higher mean bone age than males [6]. Similarly, Moca et al. (2021) conducted a retrospective study of 252 pediatric patients aged 6–15.9 years in Romania, revealing that chronological age differences between skeletal stages became statistically significant at cervical stage (CS) 4; females were more frequently found at higher cervical stages (CS5-6) [7]. Typically, CS1 and CS2 are considered prepubertal, CS3 and CS4 circumpubertal, and CS5 and CS6 postpubertal [8]. This staging system allows orthodontists to determine the optimal timing for growth modification therapy. Evidence suggests that prepubertal orthopedic treatment of Class III malocclusion can effectively induce favorable skeletal changes in both the maxilla and mandible, whereas treatment at puberty is effective at the mandibular level only [9].

An orofacial muscle imbalance is a significant contributing factor to malocclusion and skeletal growth discrepancies [10]. In Class III malocclusion patients, these dysfunctional patterns include a lower and more retruded tongue posture [11], reduced perioral muscle forces [12], and altered masticatory muscle function [13]. Myofunctional training with soft intraoral prefabricated myofunctional appliances is an emerging approach in orthopedic treatment, aimed at correcting orofacial muscle imbalances and establishing equilibrium between the cheek muscles, lips, and tongue. Eliminating such muscle imbalances can effectively improve the treatment outcomes and the long-term stability of a malocclusion correction. Additionally, the intraoral design promotes better compliance compared to extraoral devices, making them particularly suitable for growing patients in the mixed dentition phase. Among the various myofunctional appliances, the functional education appliance (EF) (OrthoPlus, Igny, France) and myobrace (MRC) (Myofunctional Research Co., Helensvale, QLD, Australia) are contemporary prefabricated myofunctional appliances that help establish proper occlusal relationships. Their standardized designs and ease of use make them valuable tools for both orthodontists and patients. However, most of the existing literature has focused on their use in the management of Class II malocclusion. Therefore, the aim of this case report is to investigate the effectiveness of prefabricated myofunctional appliances in the treatment of Class III malocclusion.

## 2. Case Presentations

### 2.1. Patient Information and Clinical Findings

**Figure 1:**
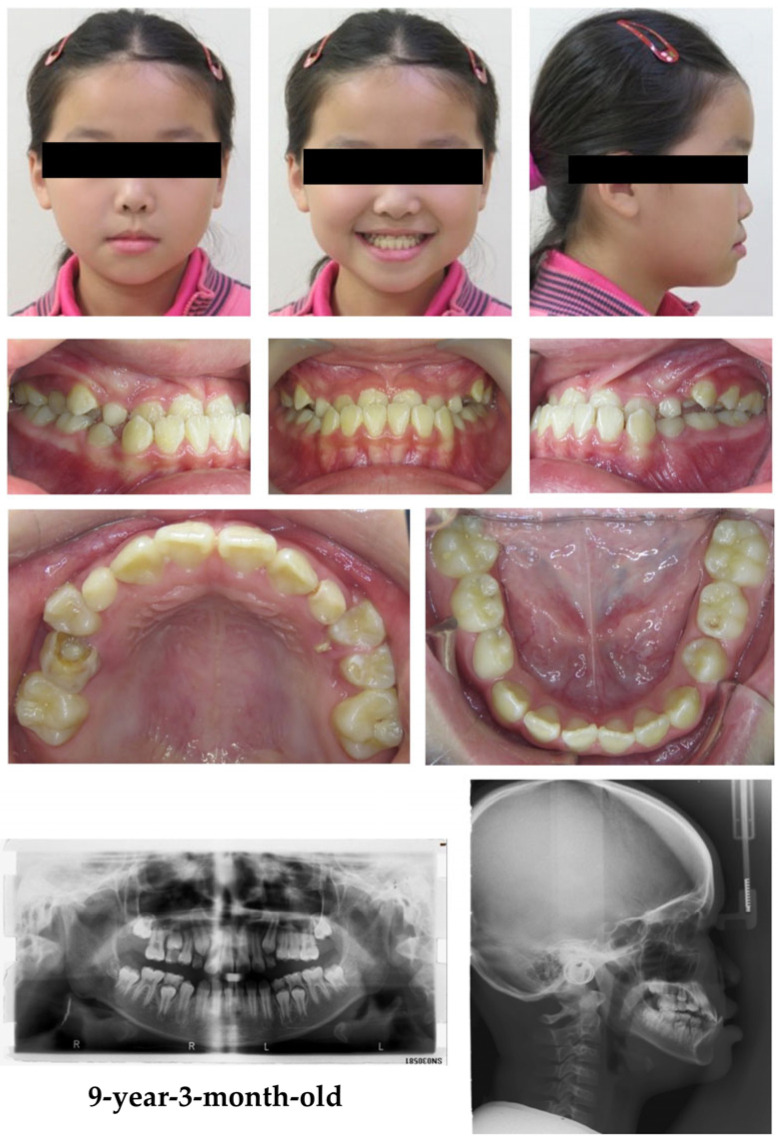
Pretreatment facial and intraoral photographs, panoramic radiograph, and cephalometric radiograph of a 9-year-3-month-old patient. The facial and intraoral photographs showed a protruding lower lip, reverse overjet, and a concave profile. The panoramic radiograph demonstrated mixed dentition, and the cephalometric radiograph indicated skeletal class III malocclusion and cervical vertebral maturation stage 2.

A 9-year-old girl consulted for orthodontic treatment due to an obvious crossbite and deep bite over the anterior arch without a functional shift. No evidence of mouth breathing, swallowing problems, or poor oral habits was observed in the patient. The frontal full smile photograph showed a negative overjet (−4 mm), while the lateral profile showed a concave pattern with a protruding lower lip. The lower dental midline was shifted 1.5 mm to the right side relative to the facial midline. Intraoral photographs showed a bilateral angle Class III molar and canine relationship and a posterior physiological openbite over the left premolar area. A panoramic radiograph showed mixed dentition and an asymmetrical mandibular ramus length. The cephalometric radiograph indicated that the patient was in CS2 (Figure 1).

### 2.2. Treatment

**Figure 2:**
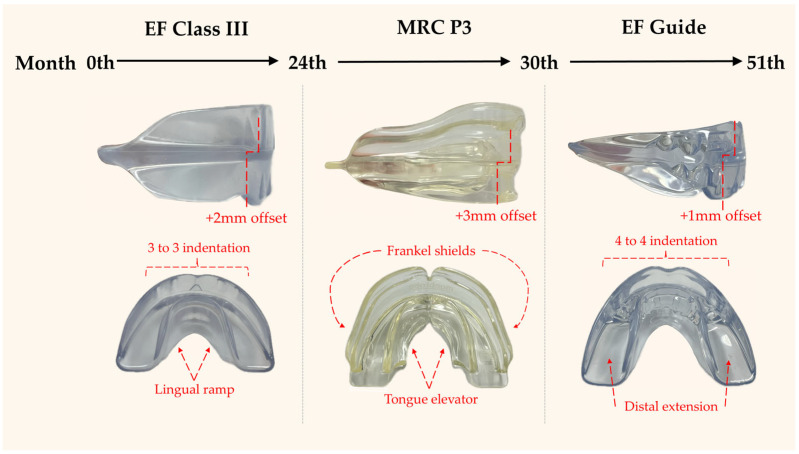
Treatment appliance sequence. The EF Class III appliance was delivered as the primary modality for 24 months. MRC P3 was further prescribed for 6 months due to the discrepancy between the arch size and the EF Class III appliance. The EF Guide was used for interdigitation and retention for 21 months. Functional design of prefabricated myofunctional appliances: lingual ramp and tongue elevator guide the tongue into its proper resting and functional position; Frankel shields for upper arch development; and indentations on the maxilla and mandible enhance dental alignment, midline correction, and retention. Abbreviations: EF—functional education appliance (OrthoPlus, Igny, France)—and MRC—myobrace (Myofunctional Research Co., Helensvale, QLD, Australia).

**Figure 3:**
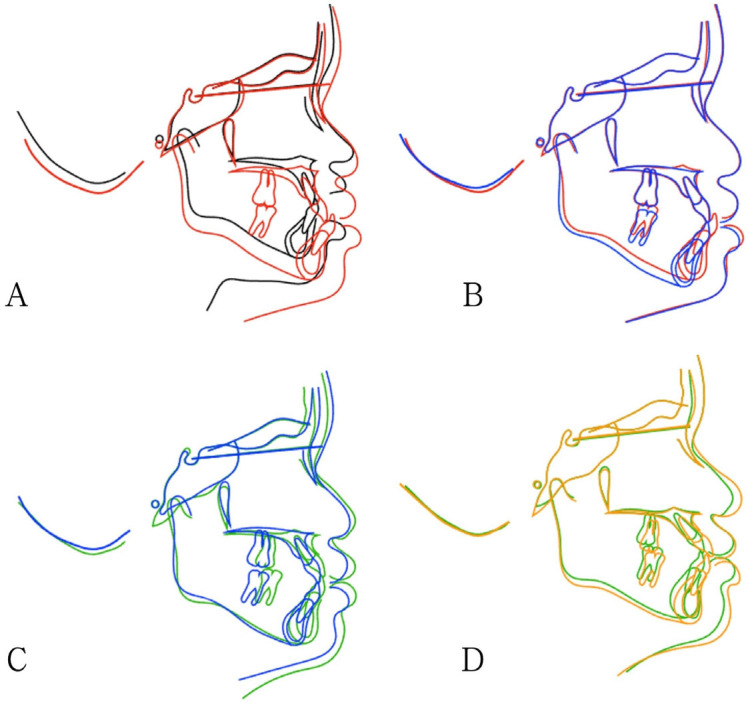
Cephalometric superimposition across treatment stages. (**A**) 5-year-old (black) to pretreatment at 9-year-old (red) showing minor anteroposterior and major vertical mandibular growth. (**B**) Pretreatment at age 9 (red) to 6 months of treatment (blue) showing clockwise mandibular rotation. (**C**) Six months (blue) to thirty months of treatment (green) showing increased maxillary and mandibular length (Class I growth pattern). (**D**) Thirty months (green) to fifty-one months of treatment (orange) showing a tendency to return to the original growth pattern of the patient with dental compensation, including flared upper incisors.

**Figure 4:**
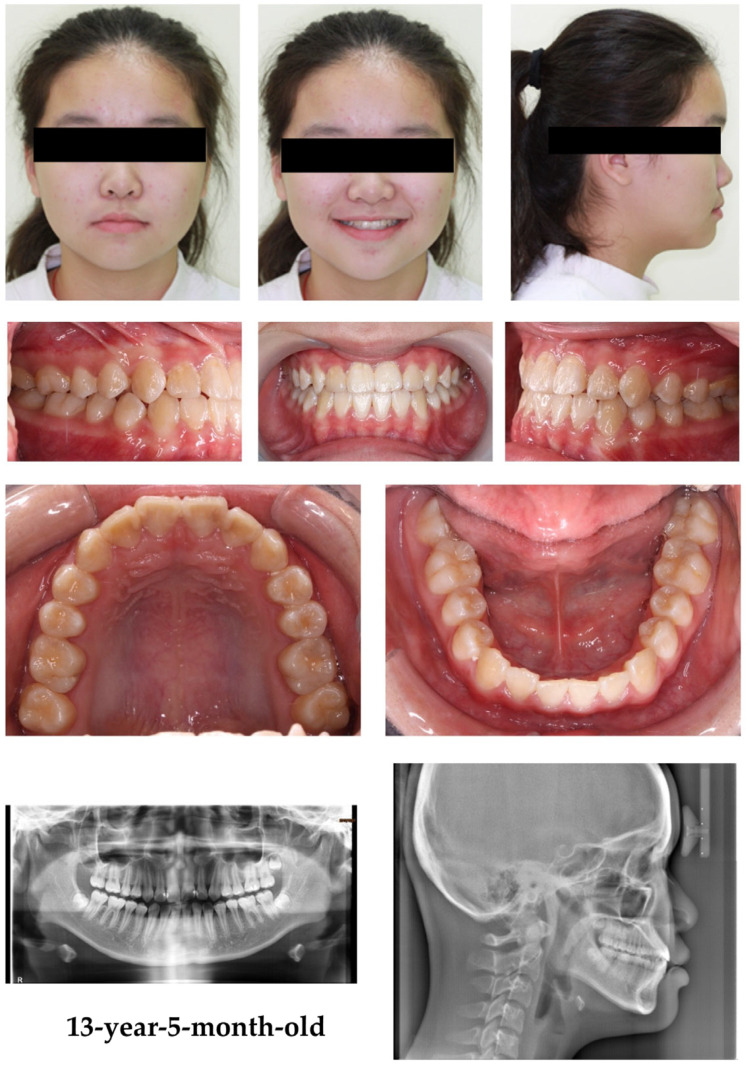
Facial and intraoral photographs, panoramic radiograph, and cephalometric radiograph at 51 months treatment time of a 13-year-5-month-old patient. The facial photographs showed a straight profile, and the intraoral photographs demonstrated acceptable overjet and overbite. The cephalometric radiograph indicated cervical vertebral maturation stage 4.

The patient declined the use of extraoral appliances, such as a facemask, and refused a palatal expander. The Class III EF was therefore delivered as the primary treatment modality, to be worn nightly and for an additional two hours during the day, accompanied by muscle, swallowing, and breathing exercises. Patient’s compliance was assessed as moderate. Operative dentistry, extraction, and oral hygiene instruction were addressed before we performed the early intervention. Periodic follow-ups were scheduled at three-month intervals. At twenty-four months (24 M) of treatment, the overjet had improved to +1.5 mm, and the overbite had improved to 1 mm. Due to the discrepancy between the arch size and the EF series appliance, the MRC P3 was prescribed for continuous Class III force application. Six months later (30 M), based on the patient’s height records and menarche timing, her growth status was determined to be close to the turning point for stable growth. Therefore, the EF Guide was used for further interdigitation and retention (Figure 2). Cephalometric superimposition (Figure 3) and analysis (Table 1) were performed to evaluate skeletal and dental changes at pretreatment (ages 5 and 9) and at post-treatment intervals (6, 30, and 51 months). Twenty-one months later (51 M), follow-up records including photographs and radiographs were also obtained (Figure 4).

### 2.3. Treatment Outcome

The comparison of cephalometric radiographs was taken at pretreatment and post-treatment (Figure 3). Figure 3A revealed that the anterior crossbite and malocclusion observed at age 5 (black line) persisted during the transition to mixed dentition at age 9 (red line) without intervention. Mandibular superimposition showed that mandibular growth was minor in the anteroposterior direction and major in the vertical direction. The increased *Y*-axis angle (Table 1) showed a vertical growth tendency.

After 6 months of treatment (blue line), the mandible was positioned downward and backward (treatment effect—clockwise rotation of mandible, Figure 3B). The overjet was corrected to an edge bite, accompanied by increased ANB (point A–nasion–point B), SN-MP (between sella–nasion and mandibular plane), and FMA (Frankfort horizontal–mandibular plane) angles (Table 1).

After 30 months of treatment (green line), the anterior crossbite was corrected, with increased SNA (between the sella, nasion, and point A) angle and maxillary length. Mandibular growth was restrained, and the SNB (between the sella, nasion, and point B) angle remained unchanged (Figure 3C and Table 1). This showed a Class I growth pattern following the short-term treatment.

After 51 months of treatment (orange line), the skeletal discrepancy between the maxilla and mandible decreased. However, the skeletal Class III pattern persisted, indicating a tendency to return to the original growth pattern of the patient. Dental compensations were observed, including flared upper incisors, an acceptable overjet and overbite, and retroclined and mildly crowded lower incisors (Figure 3D and Figure 4, Table 1).

The facial profile improved from a mildly concave to a straight profile, with forward maxillary growth and restrained mandibular growth in the sagittal direction. Furthermore, an acceptable overjet and overbite were established with dental compensation of the upper incisor proclination and lower incisor inclinations at 51 months of treatment (Figure 4).

## 3. Discussion

Class III malocclusion is one of the most challenging conditions to manage in growing patients due to its multifactorial etiology and unpredictable growth pattern. A Class III malocclusion becomes more complex when accompanied by skeletal discrepancies. Pediatric dentists must recognize these discrepancies and understand the appropriate treatment options and timing to effectively minimize them. The mixed dentition period, typically occurring between ages 6–12 years, represents an optimal time for Class III malocclusion management. It has been reported that the early mixed dentition period is the best time to begin class III treatment [14], as the circummaxillary sutures are smooth and broad at the eruption of the maxillary incisors, but will become more interdigitated in the later juvenile and early adolescent stages [15,16]. Early intervention during this period can maximize skeletal correction and guide growth in a favorable direction.

Several Class III treatment modalities have been reported, ranging from dentoalveolar camouflage to orthopedic and surgical approaches. Orthodontic camouflage typically involves proclining the maxillary incisors and retroclining the mandibular incisors to achieve an acceptable overjet and overbite. While this approach can improve occlusion, it does not address the underlying skeletal discrepancy. Moreover, factors such as skeletal severity, incisor angulation, soft-tissue profile, and periodontal health must be carefully considered during a case selection [17]. Maxillary expansion, including rapid maxillary expansion (RME), is widely used to facilitate maxillary protraction by disrupting the system of sutures. The alternate rapid maxillary expansion and constriction (Alt-RAMEC) protocol was introduced by Liou and Tsai in 2005, consisting of weekly alternating expansion and constriction over several weeks. This protocol has been reported to improve the efficiency of maxillary protraction compared to RME alone [18]. The facemask (FM) is another common orthopedic strategy that can be combined with removable appliances or RME. It has been reported that RME/FM treatment is effective in improving sagittal skeletal relationships, primarily through maxillary protraction, with favorable changes in overjet and molar relationships. In the long term, these improvements remain stable, largely due to favorable mandibular changes, and the prevalence of treatment failure is markedly lower [19]. However, these approaches often require high compliance or invasive procedures, which may limit their acceptability in young patients.

In contrast, prefabricated myofunctional appliances such as EF and MRC have emerged as less invasive alternatives for managing malocclusion in growing patients. These appliances are conducive to myofunctional training. Their use is supplemented by breathing and orofacial muscle exercises, following protocols provided by clinical dentists to normalize muscle tone in the neutral zone, reduce muscular interference, and passively guide the tongue to its proper position. The effectiveness of these appliances mediates through the neuromuscular adaptation, as demonstrated in electromyographic studies showing observable changes in the activity of key masticatory muscles such as the temporalis, masseter, and pterygoid muscles in myofunctional orthodontics [20]. In addition, the buccinator mechanism plays a crucial role in maintaining arch form and tooth position [21]. Evidence from fixed functional appliances has also shown that functional orthopedic forces can trigger motor reprogramming and postural changes, leading to a growth response [22]. These neuromuscular adjustments help establish functional balance, thereby contributing to favorable skeletal and dentoalveolar corrections.

Prefabricated myofunctional appliances, like other orthopedic treatments, combine both skeletal and dentoalveolar treatment effects. Overjet correction is achieved not only through a reduction in the maxilla–mandible discrepancy but also through upper incisor flaring, lower incisor retroclination, and mandibular rotation. It should be noted that the success of orthopedic treatment is not determined solely by the treatment appliance but is also influenced by the patient’s craniofacial growth pattern. Despite these established treatment mechanisms, most of the literature on prefabricated myofunctional appliances has focused on their use in treating Class II malocclusions, while evidence for their effectiveness in managing Class III malocclusions are relatively rare, with reports including the application of a Frankel-III-shaped prefabricated functional appliance (NOA™-F3) in a skeletal Class III patient with unilateral scissor bite and anterior crossbite, where the device opened the reverse bite and guided backward mandibular movement [23]; the treatment of a 7-year-old boy with MRC i-3N/i-3R/i-3H protocols that successfully corrected anterior and posterior crossbite, improved facial profile, and achieved Class I molar relation [24]. Although these reports provide valuable insights, the overall evidence remains limited, highlighting the need for further research on Class III cases.

In our case, the Class III EF setup included the +2 mm overjet for crossbite over-correction and guiding the mandibular position backward. After six months of treatment, a “chin cup effect” was observed, with downward and backward mandibular rotation confirmed by the increases in ANB, SN-MP, and FMA angles. This mechanism demonstrated the potential ability of Class III EF to influence vertical facial dimensions. After 30 months of treatment, the anterior crossbite was corrected, accompanied by increased SNA angle and maxillary length, indicating favorable maxillary growth. In contrast, mandibular growth was restricted with an unchanged SNB angle, demonstrating that the prefabricated myofunctional appliances can effectively restrain mandibular development. This differential growth pattern resulted in the establishment of a Class I relationship. However, this favorable Class I growth pattern was considered to represent a temporary initial effect of treatment rather than a permanent correction. After 51 months of treatment, the skeletal discrepancy between the maxilla and mandible had improved compared to the pretreatment. Despite this improvement, the skeletal Class III pattern persisted, although the corrective force continued to be applied to the maxillomandibular complex, indicating a tendency to return to the patient’s original growth pattern. This persistence of the underlying skeletal relationship necessitated reliance on dental compensation to achieve the final occlusion, suggesting that while treatment was effective in reducing the severity of the discrepancy, the capacity for permanent skeletal modification is limited, and the fundamental skeletal growth pattern of the patient remained unchanged. Considering the patient’s young age and the conflicting findings on lower third molars’ effect on anterior crowding [25,26], we do not recommend extracting the wisdom teeth at this stage. Nevertheless, extraction may be considered in future follow-up depending on the patient’s development.

Our findings align with previous reports demonstrating that prefabricated myofunctional appliances can induce backward mandibular rotation and favorable dentoalveolar compensations [23,24], which play an essential role in the correction of anterior crossbite and improvement of skeletal discrepancy. Moreover, our results demonstrated forward maxillary displacement, as evidenced by the increased SNA angle, highlighting an additional treatment effect that may further enhance skeletal correction in Class III patients. Importantly, the patient achieved clinically acceptable occlusion despite moderate compliance and refusal of extraoral devices or palatal expansion, underscoring the potential of prefabricated myofunctional appliances as a practical and less invasive treatment option in such cases.

Although the treatment outcome was acceptable to the patient and her father, certain limitations were observed, including minor issues with alignment, leveling, rotation, midline deviation, and compensated incisor positions (U1/SN and L1/MP), which may require further orthodontic treatment in the future. Moreover, the potential relapse and long-term instability remain concerns. Therefore, regular follow-up is essential to monitor stability, and in some cases, orthognathic surgery may be required. Future studies with long-term evaluation are recommended to clarify the stability of treatment outcomes with prefabricated myofunctional appliances.

## 4. Conclusions

Prefabricated myofunctional appliances represent an effective treatment option for pediatric patients with skeletal Class III malocclusion. A vertical mandibular growth pattern is conducive to correcting small-scale skeletal Class III discrepancies in patients with a normal or low mandibular plane angle. When applied during optimal growth phases, these appliances can produce favorable skeletal and dentoalveolar changes that help reduce Class III discrepancies.

## Figures and Tables

**Table 1:** Cephalometric radiograph analysis results.

	5 Years Old	Pre-Treatment (9 Years Old)	6 Months Treatment	30 Months Treatment	51 Months Treatment
SNA (°)	81	82.5	82.5	86.5	87
SNB (°)	86	87.5	85	87.5	89
ANB (°)	−5	−5	−2.5	−1	−2
SN-MP (°)	31.5	32	34	29	28.5
FMA (°)	28	31	35	27.5	27
U1 to NA (mm)	2	7	7	6.5	10
U1 to SN (°)	100	112.5	112.5	118	123
L1 to NB (mm)	1.5	5	5	3	4.5
L1 to MP (°)	87	93.5	91	90	90
Upper gonial angle (°)	56	53.5	50	52	51
Lower gonial angle (°)	72	72.5	73.5	73	73
*Y*-axis (°)	58	62.5	66	61	61.5
Cranial flexure (°)	124	123	123	125	125
CVM stage	I	II	II	III	IV

Abbreviations: SNA—the angle formed between the sella, nasion, and point A; SNB—the angle formed between the sella, nasion, and point B; ANB—the difference between the SNA and SNB; SN-MP—the angle between the sella—nasion and mandibular plane; FMA—the angle between the Frankfort horizontal and the mandibular plane; U1 to NA—the distance from the incisal edge of the upper incisor to the nasion—point A line; U1 to SN—the angle between the long axis of the upper incisor and the sella—nasion plane; L1 to NB—the distance from the incisal edge of the lower incisor to the nasion—point B line; L1 to MP—the angle between the long axis of the lower incisor and the mandibular plane; *Y*-axis—the angle between the sella—gnathion line to the Frankfort horizontal plane; and CVM—cervical vertebrae maturation.

## Data Availability

The original contributions presented in this study are included in the article. Further inquiries can be directed at the corresponding author.
